# Body condition score, morphometric measurements and estimation of body weight in mature Icelandic horses in Denmark

**DOI:** 10.1186/s13028-016-0240-5

**Published:** 2016-10-20

**Authors:** Rasmus B. Jensen, Signe H. Danielsen, Anne-Helene Tauson

**Affiliations:** Department of Large Animal Sciences, Faculty of Health and Medical Sciences, University of Copenhagen, Grønnegårdsvej 3, 1870 Frederiksberg C, Denmark

**Keywords:** Body condition score, Obesity, Icelandic horse, Overweight

## Abstract

**Background:**

Obesity is related to the development of several diseases like insulin resistance and laminitis in horses. The prevalence of obesity among mature Icelandic horses in Denmark has not been investigated previously. This study aimed to find the prevalence of obesity, to compare body condition score (BCS) based on owner perception with that of an experienced person and to correlate the BCS to body weight (BW) and morphometric measures in a group of mature Icelandic horses in Denmark. A total of 254 Icelandic horses (≥4 years; 140 geldings, 105 mares, 9 stallions) from 46 different farms were included. All horses were assigned a BCS on a scale from 1 to 9 (1 is poor, 5 is moderate and 9 is extremely fat) by their owner and by an experienced person. Two weight tapes were used to assess BW. Girth circumference (GC), neck circumference (NC) and height at withers (HW) were measured, and the GC:HW and NC:HW ratios were calculated.

**Results:**

Categorising the horses into four groups, 5.9 % were underweight (BCS 3–4), 70.1 % were optimal (BCS 5–6), 13.8 % were overweight (BCS 7) and 10.2 % were obese (BCS 8–9). The GC:HW and NC:HW ratios increased with increasing BCS, as did the BW estimated with the weight tapes. A GC:HW ratio >1.21 might indicate overweight or obesity in Icelandic horses. Horse owners underestimated the BCS of their horses compared to an experienced person.

**Conclusions:**

The results from this study show that 24.0 % of mature Icelandic horses in Denmark are overweight or obese, and that owners tend to underestimate the BCS of their Icelandic horses. The GC:HW ratio might indicate overweight or obesity, however, the ratio for Icelandic horses is different than reported for horses and ponies of other breeds.

## Background

Different subjective methods have been used to evaluate body fat accumulation in horses and ponies [1, 2], and the most commonly used is the 9-point Henneke body condition score (BCS) system originally developed for use in Quarter horse broodmares, where BCS is categorized on a scale from 1 (poor) to 9 (extremely fat) [2]. A horse or pony can be classified as overweight with a BCS of 7 and obese when the BCS is ≥8 on the Henneke scale [2]. The BCS is a subjective measure of fat deposition, and it has been suggested that morphometric measures like girth circumference:height at withers ratio (evaluating overall adiposity) and neck circumference:height at withers ratio (apparent neck adiposity) can be used as an objective method [3].

Equine obesity is considered the most important welfare issue affecting equines in the western world [4], and obesity is related to an increased risk of insulin resistance [5] and laminitis [6] in horses and ponies. The prevalence of overweight and obesity has been reported to be 45 % in riding horses (n = 319) in Scotland [7], 27 and 35 % in a population of leisure horses (n = 96) in UK during winter and summer [8] and 51 % in a group of mature light-breed horses (n = 300) in USA [9]. In studies using owner reported BCS the prevalence of obesity has been found to be 21 % in a population of horses (n = 158) in the UK [10] and 31 % in a larger group of horses and ponies (n = 792) in the UK [11]. However, it has been reported that owners might underestimate the BCS of their horses and the actual prevalence of obesity might have been higher than reported in those studies [7, 10].

The Icelandic horse is considered to be an “easy keeper”, i.e. easy to keep in a good body condition [12], and overweight and obesity is reported by horse owners to be a common problem in this breed. However, the prevalence of overweight and obesity among mature Icelandic horses in Denmark has not been investigated previously.

This study aimed (1) to find the prevalence of overweight and obesity among mature Icelandic horses in Denmark, (2) to compare BCS based on owner perception with that of an experienced person, and (3) to relate BCS to morphometric measurements and body weight (BW).

## Methods

### Experimental design and animals

The study was designed as a cross sectional study. All local riding clubs for Icelandic horses in Denmark were contacted by email with a letter explaining the aim and criteria for participation in this study (purebred, leisure or sports horse, ≥4 years old and healthy according to the owner), and horse owners volunteered to participate in the study. A total of 254 mature Icelandic horses (140 geldings, 105 mares and 9 stallions) from 46 different farms covering most of Denmark (Jutland, Funen and Zealand) having 1–22 horses per farm (mean 6 ± 5 horses) was included in this study. The age of the horses was between 4 and 26 years (mean 11.8 ± 5.4 years).

### Data collection

Data was collected during 3 consecutive weeks during the end of November to the start of December. All horses were assigned a BCS according to the Henneke scale [9] from 1 (poor) to 9 (extremely fat) by an experienced person, and a group of horses (n = 216) were also assigned a BCS based on their owners perception. The same experienced person gave BCS to all horses and the owners were briefly introduced to the Henneke scale before they assigned their own horse a BCS.

Morphometric measures were taken to support the subjective BCS with objective measurements. Height at withers (HW) (in cm), girth circumference (GC) (in cm) and neck circumference (NC) (in cm) halfway between the poll and the withers were measured once on each horse, and the NC:HW and the GC:HW ratios were calculated [3].

Two weight tapes (Weight tape 1: Dodson & Horrell Ltd, Kettering, UK; Weight tape 2: Virbac Equimax, Milperra NSW, Australia) were used to estimate body weight (BW) of all the horses.

### Calculations and statistical analyses

All statistical analyses were performed in SAS^®^ (SAS^®^ version 9.4, SAS institute Inc. Cary, North Carolina, USA). The prevalence of horses with a BCS from 1 to 9 was calculated based on the measurements from the experienced person, and Kappa statistics and Spearman rank correlation coefficient was used to evaluate the agreement between BCS based on the experienced person and the horse owners. The effect of age, sex and farm on BCS was analysed using the MIXED procedure in SAS^®^. All horses were grouped into four categories of either underweight (BCS 3–4), optimal (BCS 5–6), overweight (BCS 7) or obese (BCS 8–9). The effect of age, HW, GC, NC, GC:HW ratio, NC:HW ratio and estimated BW on BCS category was analysed using the MIXED procedure in SAS^®^. Results are presented as least square means with their 95 % confidence intervals. Effects were considered significant if P < 0.05. Additionally, a Bland–Altman plot [13] was used to evaluate the two different weight tapes, where the difference between the paired measurements was plotted against the average of the two measurements.

## Results

### Body condition score

**Fig. 1:**
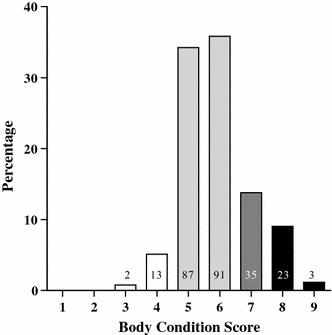
The percentage of Icelandic horses (n = 254) with a body condition score (BCS) of 1–9. The* numbers* at the *bars* indicate the number of horses with the given BCS

**Fig. 2:**
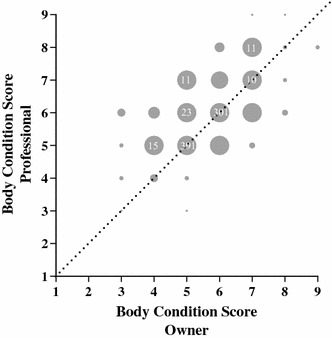
The body condition score (BCS) assigned by an experienced person (*y* axis) plotted against the BCS assigned by the horses owners (*x* axis). The size of the *circles* indicates the number of observations, and if there are more than ten observations, then the actual number is given in the *circles*

Body condition scores of the 254 horses varied from 3 to 9 (Fig. 1). There was no effect of age (P > 0.10) or sex (P = 0.10) on the BCS, but farm had an effect (P < 0.001) (data not shown). Of the 254 horses 216 were also given a BCS by their owners. The BCS assigned by the owners correlated with the BCS assigned by the experienced person with a Spearman rank correlation coefficient of 0.59 (P < 0.001), whereas the kappa statistics was 0.21 showing a poor agreement between the horse owners and the experienced person. Generally, horse owners underestimated (90/216) the BCS of their own horses more than they overestimated the BCS (41/216) compared to an experienced person, as shown in Fig. 2. When the horses were categorized into the four groups, 5.9 % of the horses were underweight (BCS 3–4), 70.1 % were optimal (BCS 5–6), 13.8 % were overweight (BCS 7) and 10.2 % were obese (BCS 8–9) (Table 1).

**Table 1:** Categorisation of Icelandic horses in relation to body condition score

	Body condition score category				
	Underweight (BCS 3–4)	Optimal (BCS 5–6)	Overweight (BCS of 7)	Obese (BCS 8 or 9)	P value
Number of horses	15	191	35	26	–
Percentage of horses, %	5.9	70.1	13.8	10.2	–
Age, years	12.5 (8.7–16.3)	10.8 (9.3–11.7)	11.1 (9.2–13.0)	9.9 (7.6–12.1)	NS
Height at withers, cm	136 (134–138)	137 (137–138)	136 (135–137)	137 (136–138)	NS
Girth circumference, cm	158 (156–161)^d^	166 (166–167)^c^	172 (171–174)^b^	178 (176–180)^a^	<0.001
Neck circumference, cm	83.1 (79.9–86.4)^d^	89.1 (88.1–90.0)^c^	92.0 (89.9–94.1)^b^	95.7 (93.3–98.2)^a^	<0.001
Girth circumference:height at withers ratio	1.16 (1.14–1.18)^d^	1.21 (1.21–1.22)^c^	1.27 (1.25–1.28)^b^	1.29 (1.28–1.31)^a^	<0.001
Neck circumference:height at withers ratio	0.61 (0.59–0.63)^c^	0.65 (0.64–0.66)^b^	0.68 (0.66–0.69)^a^	0.70 (0.68–0.72)^a^	<0.001
Estimated body weight: tape 1, kg	310 (295–325)^d^	355 (350–359)^c^	390 (380–399)^b^	423 (412–435)^a^	<0.001
Estimated body weight: tape 2, kg	328 (311–346)^d^	374 (369–379)^c^	412 (401–423)^b^	444 (431–457)^a^	<0.001

The effect of age (years), height at withers (cm), girth circumference (cm), neck circumference (cm), girth circumference:height at withers ratio, neck circumference:height at withers ratio and body weight (kg) estimated with the two weight tapes on BCS category

*NS* non-significant

^a,b,c,d^Values in the same row without common superscripts differ significantly (P < 0.05)

### Morphometric measurements and body weight estimation

**Fig. 3:**
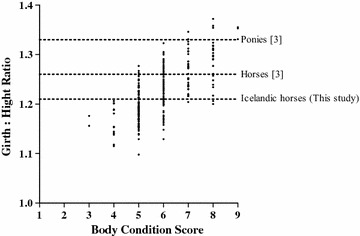
The girth circumference:height at withers ratio plotted against the body condition score (BCS). The *dotted lines* indicate the cut off values for ponies and horses indicating overweight or obesity (BCS ≥ 7) [3] and the value suggested for Icelandic horses in this study

**Fig. 4:**
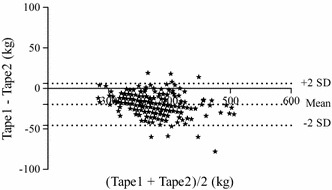
Bland-Altman plot of the agreement between the two weight tapes used to estimate body weight. The *y* axis denotes the difference between the two weight tapes (mean ± 2SD: −19.8 ± 26.5 kg), and the *x* axis the average of the two weight tapes. The *dotted lines* represent the 95 % confidence interval

Age and height did not affect the BCS category as shown in Table 1. However, GC, NC and GC:HW ratio all increased (P < 0.001) as BCS increased from one category to another (Table 1). The NC:HW ratio increased when the BCS increased (P < 0.001), but there was no difference between a BCS 7 and a BCS of 8–9 (Table 1). The GC:HW ratio in relation to BCS is shown in Fig. 3. The estimated BW with weight tape 1 and weight tape 2 was affected by BCS category and increased as BCS increased (Table 1). However, the Bland–Altman plot (Fig. 4) showed that weight tape 2 systematically estimated the BW of the horses 19.8 kg higher than weight tape 1.

## Discussion

The main objective of this study was to find the prevalence of overweight and obesity among Icelandic horses in Denmark. This study is the first to document that overweight and obesity is a common problem among Icelandic horses in Denmark and that 24 % of the horses in this study were overweight or obese. This relatively high prevalence of overweight and obesity has also been reported in other studies where the prevalence varied from 27 to 45 % [4–6]. This study did also find that horse owners were more likely to underestimate the BCS of their horses compared to an experienced person, and this has also been highlighted in other studies [4, 7].

A BCS system is a subjective measure of body fat accumulation, and the original 9-point Henneke BCS system was originally developed for use in Quarter horse broodmares [2]. However, it has been used frequently in studies using different breeds where the BCS has been used to describe the fatness of the animals. A 5 point BCS system is commonly used in Iceland [14], however, the original paper is only available in Icelandic and there are no peer reviewed papers explaining or validating this system. One reason for horses being overweight or obese might be that owners underestimate the BCS as highlighted above. However, a poor understanding of the different BCS systems used in practise and in different studies might also be a reason for the different results. The horse owners were only briefly introduced to the BCS systems and had no guidance when assigning a BCS in this study nor in other studies [4, 7].

Weighing horses is the most accurate method to detect fluctuations in BW, but a weigh bridge is expensive and often not available on farms and weight tapes are commonly used as a proxy. The two different weight tapes compared in this study were able to differentiate the BCS categories and increasing BCS did also increase the estimated BW. However, it was clear from the Bland–Altman plot that weight tape 2 overestimated the BW by approximately 20 kg compared to weight tape 1. The accurate BW might be of special importance when providing different medication to horses based on BW. In a small group of Icelandic horses (n = 13) the actual BW was compared to estimated BW using a weight tape (Pfizer A/S, Hvidovre, Denmark) or a formula using girth circumference and body length [1], and it was found that BW can be estimated from measurements of GC and body length, and that weight tapes seem to be a suitable method. However, it requires more research to document which weight tapes and formulas are most appropriate for Icelandic horses and if other morphometric measures like body length need to be included.

The GC:HW ratio and the NC:HW ratio have been suggested as morphometric measurements that would indicate overweight or obesity [3], and a horse or pony could be considered overweight (BCS ≥ 7) with a GC:HW ratio ≥1.26 for horses or 1.33 for ponies. However, these cut off values are not suitable for Icelandic horses as shown in Fig. 3. A value >1.21 for the GC:HW ratio might be appropriate for Icelandic horses. Morphological differences between breeds result in different GC:HW ratios making comparisons of ratios between breeds difficult. In this study 88 out of 191 Icelandic horses with a BCS of 5 or 6 did have a GC:HW ratio >1.21 (Fig. 3), hence the GC:HW ratio should only be considered as an indication of overweight or obesity.

Neck crest adiposity has been described using the cresty neck score, where a score of 0 is no visual appearance of a crest and 5 is a crest so large that it permanently drops to one side [3]. This score was unfortunately not assigned to the Icelandic horses in this study, but a horse or pony may be considered having a cresty neck score of ≥3 if the NC:HW ratio is ≥0.63 for horses and ≥0.68 for ponies. In this study the NC and the NC:HW ratio increased when the BCS increased. Whether, cresty neck score and NC:HW ratio are related in Icelandic horses requires further studies.

## Conclusions

The results from this study show that 24.0 % of mature Icelandic horses are overweight or obese, and that owners tend to underestimate the BCS of their horses. A GC:HW ratio >1.21 might indicate overweight or obesity in Icelandic horses, and this ratio is different than reported for horses and ponies of other breeds.

## Abbreviations

BCS: body condition score
BW: body weight
GC: girth circumference
HW: height at withers
NC: neck circumference
